# Some Prescription Difficulties

**Published:** 1910-11-26

**Authors:** 


### Therapeutics.

### SOME PRESCRIPTION DIFFICULTIES.

From time to time there appear in the *Pharmaceutical Journal* actual prescriptions which have proved difficult to dispense; and it is always of interest to learn the reasons for the difficulties that arise. The following are a series of such prescriptions, together with the explanations given by Mr. William Duncan, Ph.C., F.C.S. :—

℞ Apomorphinæ ... ... ... ... gr. $\frac{1}{10}$

Sodii iodidi ... ... ... ... ... ʒj.

Ammonii carbonatis ... ... ... ʒij.

Spiritus chloroformi ... ... ... ʒiv.

Aquam cinnamomi ad ... ... ... ℥vj.

Misce. Fiat mistura.

This turns quite black in the bottle, and makes a very uninviting medicine. The change depends upon the action of the ammonium carbonate upon the apomorphine. The latter should be prescribed in an acid mixture. It is impossible to render the above prescription acid without decomposing the ammonium carbonate.

℞ Ammonii benzoatis ... ... ... ... ʒiij.

Potassii bromidi ... ... ... ... ʒv.

Spiritus ætheris nitrosi ... ... ... ʒiv.

Tincturæ hyoscyami ... ... ... ʒvj.

Spiritus chloroformi ... ... .... ʒiiiss.

Infusum uvæ ursi ad ... ... ... ℥vj.

Misce. Fiat mistura.

This mixture turns brown as though from liberation of free bromine. Experiments, however, show that the coloration is not due to bromine, but to pigment produced by the action of the sweet spirit of nitre upon the infusion of uva ursi. Sweet spirit of nitre can be mixed with a strong solution of potassium bromide without any evidence of bromine being liberated, either in the cold or after heating. Even after long standing it is doubtful if bromine is set free from the mixture of these two drugs. The addition of spiritus ætheris nitrosi to infusion of uva ursi, however, immediately produces a distinct coloration similar to that of free bromine; and when chloroform is added and shaken up with them, it separates out with a yellow tint, as if tinged with bromine. A dilute solution of gallic acid behaves similarly in this respect, so that the coloration of the above mixture is probably due to oxidation of the gallo-tannic acid of the uva ursi, and not to free bromine at all.

℞ Liquoris bismuthi ... ... ... ... ℥i.
Liquoris magnesii carbonatis ... ... ℥ij.
Misce. Fiat mistura.

This mixture forms a dense precipitate which consists of a combined citrate of magnesium and bismuth, together with a little carbonate. It is necessary either to prescribe mucilage to keep this precipitate in suspension, or else to advise the patient what the medicine will be like, and tell him to shake the bottle well before measuring out a dose.

℞ Quininæ sulphatis ... ... ... ... ʒss.

Acidi sulphurici diluti ... ... ... ♏x

Potassii acetatis ... ... ... ... ʒij.

Aquam ad ... ... ... ... ... ℥vj.

Misce. Fiat mistura.

This goes solid in the bottle, although it might have been thought that the acidity of the mixture would have kept the alkaloid in solution. It is not absolutely certain why it goes solid, some observers holding that the change is due to the production of quinine hydrate, others regarding it as a salting out of the quinine acetate by the potassium acetate.

℞ Codeinæ ... ... ... ... ... gr. xx.

Acidi hydrobromici ... ... ... ʒvj.

Syrupi limonis ... ... ... ... ʒvj.

Aquam cinnamomi ad ... ... ... ℥iij.

Misce. Fiat mistura.

This, like the quinine prescription just discussed, gives a dense precipitate soon after the mixture is made up. This is probably due to the salting out of the alkaloid as the bromide. The prescription is therefore not a good one; but if it is desired not to alter it, mucilage should be added to it, in order to suspend the precipitate; and the patient should be instructed to shake the bottle well before taking a dose. The syrup of lemon assists in suspending the codeine bromide, but by itself it is insufficient for the purpose.

### More Incompatibles.

℞ Sodii salicylatis ... ... ... ... ʒiss.

Acidi nitrici diluti ... ... ... ... ʒij.

Tincturæ cardamomorum compositæ ... ʒiv.

Aquam ad ... ... ... ... ... ℥vj.

Misce. Fiat mistura.

This is an example of absolute incompatibility. The druggist to whom the prescription is sent is not at liberty to neutralise the nitric acid on his own responsibility. The prescription would be referred back to the prescriber, and if he still wished it made up as it is written, the patient would require to be told to shake the bottle well before taking the medicine.

℞ Quininæ sulphatis ... ... ... ... gr. xx.

Acidi sulphurici diluti ... ... ... ℥ss.

Misce. Fiant guttæ.

This prescription might cause trouble if not carefully dispensed. The quinine needs to be added little by little to the whole of the acid, so that the acid quinine sulphate, as it is formed, may dissolve in the water of the dilute sulphuric acid.

℞ Quininæ sulphatis ... ... ... ... ʒj.

Potassii chloratis ... ... ... ... gr. xxx.

Syrupi ferri iodidi ... ... ... ℥iss.

Aquam ad ... ... ... ... ... ℥iij.

Misce. Fiat mistura.

Several mutual decompositions occur in this mixture. The potassium chlorate will oxidise the ferrous iodide, and liberate iodine according to the following equation:—

$$2FeI_2+KClO_3+3H_2O=KCl+Fe_2(HO)_6+2I_2.$$

The liberated iodine will in turn attack the quinine sulphate, forming "herapathite" or iodo-quinine.

℞ Aloin ... ... ... ... ... ... gr. xij.
Sodii bromidi ... ... ... ... ʒj.
Syrupi aurantii ... ... ... ... ℥iij.
Misce. Fiat mistura.

This mixture is sometimes yellow and at other times reddish brown, so that patients are apt to think they are not having the same medicine each time. The reason for the variation in colour is that aloin is sensitive to alkalies, and the sodium bromide contains the latter in varying quantity, causing the aloin to go a paler or a darker brown as the case may be.

℞ Sodii iodidi ... ... ... ... ... ʒiss.

Sodii bicarbonatis ... ... ... ... ʒiss.

Extracti glycyrrhizæ liquidi ... ... ℥j.

Aquam ad ... ... ... ... ... ℥iv.

Misce. Fiat mistura.

This medicine effervesces when it is dispensed, with the consequent danger of bursting the bottle if the latter is corked too soon and too tightly. The reason for the effervescence is the acidity of the liquorice extract, which varies much in different samples. Some commercial extracts are distinctly alkaline from the use of excess of ammonia to prevent precipitation. The addition of an alkali bicarbonate to galenicals sometimes causes effervescence for another reason—namely, the presence of a calcium or magnesium salt of a mono-basic acid, according to the following equation:—

$$CaAcid+2NaHCO_3=2NaAcid+CaCO_3+H_2O+CO_2.$$

### Precipitates.

℞ Liquoris bismuthi ... ... ... ... ℥j.

Sodii bicarbonatis ... ... ... ... ʒij.

Aquam ad ... ... ... ... ... ℥vj.

Misce. Fiat mistura.

This mixture may remain clear for a long time; but, on the other hand, it may cause a precipitate to form before very long. The reason for this is that saturated solutions of alkali bicarbonates and strong solutions of carbonates or hydroxides sooner or later cause decomposition of the bismuth citrate, with formation of insoluble carbonate or hydroxide.

℞ Hydrargyri subchloridi ... ... ... gr. x.

Acidi hydrocyanici (Scheele) ... ... ʒss.

Paraffini mollis }

Adipis lanæ ... } ... ... ... ... ℥ss.

Misce. Fiat unguentum.

The trouble in this prescription is that hydrocyanic acid converts calomel, the mercurous salt, into the mercuric, and a black powder which readily yields metallic mercury results. This reaction is probably common to all mercurous salts, in accordance with the following equation:—

$$Hg_2Cl_2+4HCN=H_2Hg(CN)_4+2HCl+Hg.$$

The best way of dealing with the above prescription so as to retard or prevent the interaction is to triturate the calomel with the paraffin, and the hydrocyanic acid with the wool fat, the two compounds being then mixed together to make the completed ointment.

